# Interactive visualization tools for the structural biologist

**DOI:** 10.1107/S0021889813017858

**Published:** 2013-08-03

**Authors:** Benjamin T. Porebski, Bosco K. Ho, Ashley M. Buckle

**Affiliations:** a Department of Biochemistry and Molecular Biology, Monash University, Clayton, Victoria 3800, Australia; b Monash eResearch Center, Monash University, Clayton, Victoria 3800, Australia

**Keywords:** PDB visualization, X-ray diffraction images, data management

## Abstract

In structural biology, management of a large number of Protein Data Bank (PDB) files and raw X-ray diffraction images often presents a major organizational problem. Existing software packages that manipulate these file types were not designed for these kinds of file-management tasks. This is typically encountered when browsing through a folder of hundreds of X-ray images, with the aim of rapidly inspecting the diffraction quality of a data set. To solve this problem, a useful functionality of the Macintosh operating system (OSX) has been exploited that allows custom visualization plugins to be attached to certain file types. Software plugins have been developed for diffraction images and PDB files, which in many scenarios can save considerable time and effort. The direct visualization of diffraction images and PDB structures in the file browser can be used to identify key files of interest simply by scrolling through a list of files.

## Abstract 2

Two software plugins are presented for the Mac OSX operating system that allow rapid and convenient visualization of Protein Data Bank files and X-ray diffraction images directly within the file browser, without the need for full-featured applications.

## 1. Introduction

Data visualization is an important and daily activity in structural biology, and often requires several specialized applications. The workstation of the structural biologist is littered with an excess of data, which are most frequently X-ray diffraction images and atomic structure coordinates in PDB format. Although numerous software applications exist for opening and manipulating both X-ray diffraction images [*iMOSFLM* (Battye *et al.*, 2011 ▶), *HKL-2000* (Minor *et al.*, 2006 ▶), *Adxv* (Adxv, 2013 ▶), *XDS* (Kabsch, 1988 ▶)] and Protein Data Bank (PDB; Berman *et al.*, 2000 ▶) files [*PyMOL* (Schrödinger, 2010 ▶), *Chimera* (Pettersen *et al.*, 2004 ▶), *QtMG* (McNicholas *et al.*, 2011 ▶), *VMD* (Humphrey *et al.*, 1996 ▶)], they were primarily designed to analyse files individually. When surveying a large number of files, it is cumbersome to load each file individually into a full-featured application simply to inspect the file. Instead, it would be much simpler if one could quickly search and preview files in the file browser without the execution of a full-featured application. The need for this is commonly seen with X-ray diffraction data sets, where there may be hundreds or thousands of files in a folder, and a rapid assessment of the data quality would be incredibly useful.

In regard to this, we have exploited a functionality of the Apple Macintosh operating system (OSX) called QuickLook, which allows custom-designed previews of any kind of file. By default, QuickLook allows for the visualization of text, image and video files directly from the file manager (*Finder*), without the need for full-featured software such as Microsoft *Word* or Adobe *Photoshop*. Specifically, we have developed two QuickLook plugins that allow for rapid visualizations of X-ray diffraction images and atomic protein structure files in PDB format. Furthermore, PDB files may be interacted with in three dimensions. These plugins can save a considerable amount of time and effort in browsing through large data sets.

## 2. Software design and implementation

**Figure 1:**
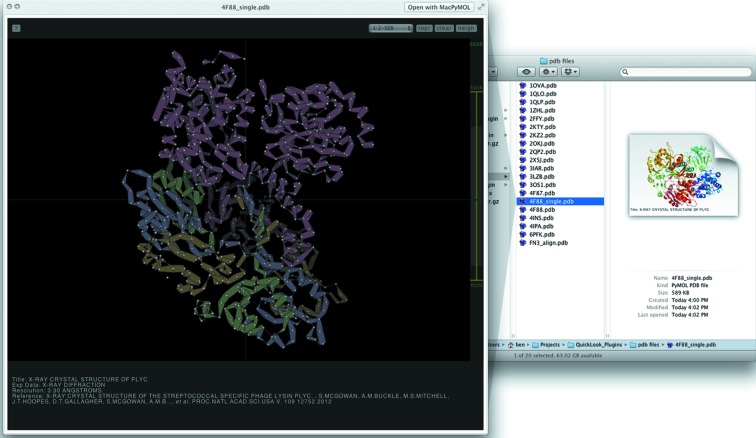
Available views of a folder containing PDB files. Shown on the right is a *Finder* window of a folder containing PDB files with the *PyMOL*-rendered cartoon thumbnail. On the left is a QuickLook popup of the same PDB file using the *Jolecule* viewer.

**Figure 2:**
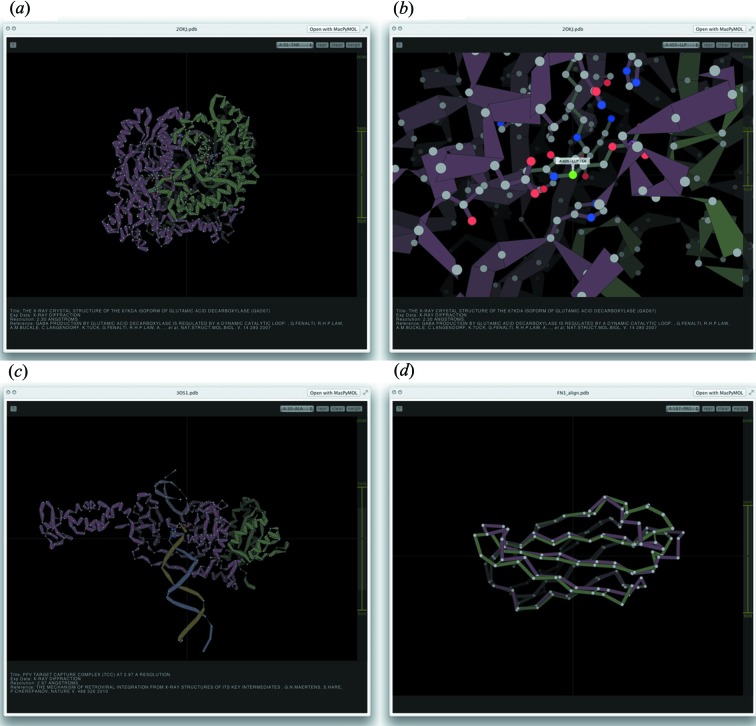
Available QuickLook views of PDB files, using *Jolecule*. (*a*) Default cartoon view of a multi-chain protein complex, showing the two chains as different colours. (*b*) Zoomed view of a protein active site showing control of the *z*-slab, labelling of a selected residue and display of neighbouring residues as ball-and-stick representation. (*c*) Cartoon view of a protein–DNA complex, showing the two protein chains as red–brown and green and the DNA helix as blue and gold. (*d*) Overlay of Cα chains of two similar structures.

We have written two independent QuickLook plugins, one for PDB files and the other for X-ray diffraction images (.osc and .img file extensions, supporting ADSC, mar/marCCD, Rigaku, CBF/MINI-CBF, Bruker and Oxford Diffraction detector/image formats). The plugin for PDB files, *QuickLookPDB*, takes advantage of the thumbnail views present in the OSX *Finder* application. *QuickLookPDB* will use, if available, an X11 version of *PyMOL* to render a cartoon-represented image of the PDB file, caching the image for future usage (Fig. 1 ▶, right). *QuickLookPDB* also offers a second interactive mode of visualization (Fig. 1 ▶, left). By pressing the spacebar, a web page is dynamically generated that renders the protein using the HTML5-based protein viewer *Jolecule* (http://github.com/boscoh/jolecule). *Jolecule* provides a number of interactions sufficient to allow a thorough examination of the protein: three-dimensional rotation and zoom, several representation styles, a distance measure, slab-view, and a selector for residues (Fig. 2 ▶). Specifically, this mode of *QuickLookPDB* leverages the feature in Mac OSX to hook into the Webkit HTML-rendering library from anywhere within the operating system. As there is a limit to the responsiveness of *Jolecule* for large mol­ecules, *QuickLookPDB* abstains from using *Jolecule* if the PDB file contains over 1.2 MB of atomic coordinates. Instead *QuickLook­PDB* will display the *PyMOL*-rendered image. Furthermore, *QuickLookPDB* at­tempts to extract useful metadata from the PDB file, such as title, experimental method used for structure determination, resolution, and a reference containing the author list and publication (if applicable). In the thumbnail view, only the title is displayed (Fig. 1 ▶), whilst all available metadata are displayed within *Jolecule* (Figs. 1 ▶ and 2 ▶). In summary, *QuickLookPDB* provides the user with a rapid preview and identification of a desired PDB file, as well as instantaneous interactivity, without leaving the file browser.

**Figure 3:**
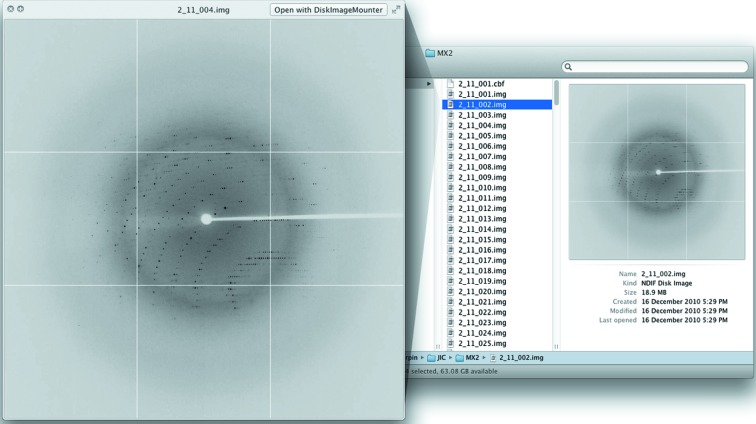
Available views of a folder containing X-ray diffraction images. Shown on the right is a *Finder* window displaying the thumbnail view of a diffraction image. On the left is the QuickLook popout of the same diffraction image.

The second plugin we have developed, *QuickLookDiffract*, allows for the quick preview of X-ray diffraction images, without the need for fully featured applications such as *iMOSFLM* (Fig. 3 ▶). *QuickLookDiffract* functions by incorporating a customized build of the *diff2jpeg* application from the *CCP4* package (Winn *et al.*, 2011 ▶). Images are rendered as JPEG files using *diff2jpeg*, cached to a temporary folder, loaded up by the plugin and displayed to the user.

**Figure 4:**
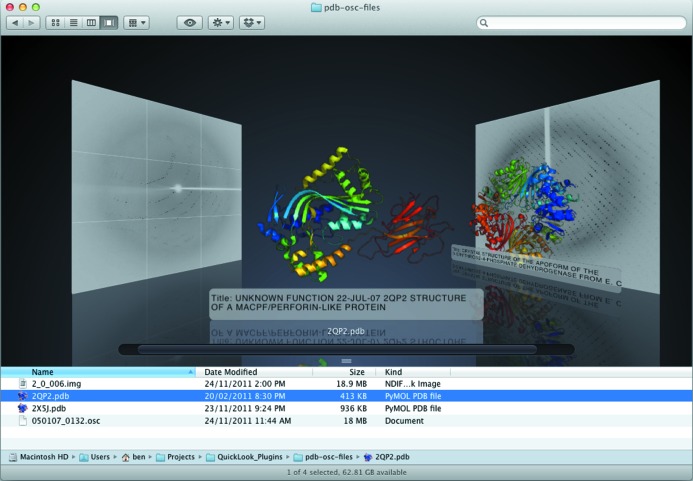
Cover Flow view of a folder containing PDBs and diffraction images.

**Figure 5:**
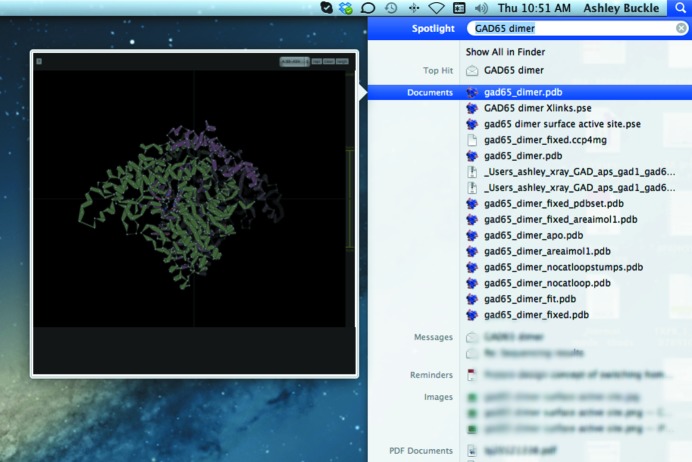
Instant three-dimensional visualization of search results using *Jolecule*.

An added utility of this software is the rapid visualization of search results. Typically, PDB files are not stored logically in an ordered file system, but are instead downloaded *ad hoc* to various folders scattered throughout the file system. Using the spotlight search function built in to Mac OSX, it is possible to visually inspect the results of a simple search (Fig. 4 ▶). Finally, another popular and useful finder view is ‘Cover Flow’ (Fig. 5 ▶). Cover Flow integrates very nicely with our plugins, showing a stack of documents in a folder and allowing the user to visually flip through the folder.

The plugins have both been tested on OSX 10.6.x, 10.7.x and 10.8.x, resulting in X-ray diffraction images for all three major versions; however, *Jolecule* is only compatible with OSX versions 10.7.x and greater, requiring WebKit to be available to QuickLook. We have made the plugins available as a binary with installer, as well as a release of the source code of each plugin and the modifications made to the *CCP4* *diff2jpeg* application. The plugin installer and source code can be downloaded from http://benporebski.github.io/CrystallographyQLPlugins/.

## 3. Conclusions

The developed plugins aim to be a useful tool for the structural biologist, allowing for rapid identification and visualization of structural data, saving time and effort during daily activities. The plugins and source code are freely available at the following link: http://benporebski.github.io/CrystallographyQLPlugins/.

General comments and suggestions for improvements are welcome and should be addressed to Ashley Buckle at ashley.buckle@monash.edu or Bosco Ho at bosco.ho@monash.edu.
